# In Vitro Multi-Bioactive Potential of Enzymatic Hydrolysis of a Non-Toxic *Jatropha curcas* Cake Protein Isolate

**DOI:** 10.3390/molecules29133088

**Published:** 2024-06-28

**Authors:** Olloqui Enrique Javier, González-Rodríguez Maurilio Alejandro, Contreras-López Elizabeth, Pérez-Flores Jesús Guadalupe, Pérez-Escalante Emmanuel, Moreno-Seceña Juan Carlos, Martínez-Carrera Daniel

**Affiliations:** 1 Centro de Biotecnología de Hongos Comestibles, Funcionales y Medicinales (CB-HCFM), Colegio de Postgraduados, Campus Puebla, Boulevard Forjadores de Puebla no. 205, Puebla 72760, Mexico; 2 Área Académica de Química, Instituto de Ciencias Básicas e Ingeniería, Universidad Autónoma del Estado de Hidalgo, Carretera Pachuca-Tulancingo km 4.5, Mineral de la Reforma, Hidalgo 42185, Mexico; 3 Departamento de Biotecnología, División de Ciencias Biológicas y de la Salud, Universidad Autónoma Metropolitana, Campus Iztapalapa, Avenida San Rafael Atlixco 186, Mexico City 09340, Mexico; 4 Colegio de Postgraduados, Campus Montecillo, Carretera Mexico-Texcoco km 36.5, Texcoco 56264, Mexico

**Keywords:** *Jatropha curcas*, bioactive peptides, antioxidant, antihypertensive, antidiabetic, alcalase, flavourzyme

## Abstract

The *Jatropha curcas* cake, a protein-rich by-product of biofuel production, was the subject of our study. We identified and quantified the ACE inhibitory, antioxidant, and antidiabetic activities of bioactive peptides from a *Jatropha curcas* L. var Sevangel protein isolate. The protein isolate (20.44% recovered dry matter, 38.75% protein content, and 34.98% protein yield) was subjected to two enzyme systems for hydrolysis: alcalase (PEJA) and flavourzyme (PEJF), recording every 2 h until 8 h had passed. The highest proteolytic capacity in PEJA was reached at 2 h (4041.38 ± 50.89), while in PEJF, it was reached at 6 h (3435.16 ± 59.31). Gel electrophoresis of the PEJA and PEJF samples showed bands corresponding to peptides smaller than 10 kDa in both systems studied. The highest values for the antioxidant capacity (DPPH) were obtained at 4 h for PEJA (56.17 ± 1.14), while they were obtained at 6 h for PEJF (26.64 ± 0.52). The highest values for the antihypertensive capacity were recorded at 6 h (86.46 ± 1.85) in PEJF. The highest antidiabetic capacity obtained for PEJA and PEJF was observed at 6 h, 68.86 ± 8.27 and 52.75 ± 2.23, respectively. This is the first report of their antidiabetic activity. Notably, alcalase hydrolysate outperformed flavourzyme hydrolysate and the cereals reported in other studies, confirming its better multi-bioactivity.

## 1. Introduction

Global use of green technologies is necessary for a sustainable future. Biofuels are an alternative to meet the demand to overcome the detrimental dependence on fossil fuels [1]. One primary biofuel source is *Jatropha curcas*, widely cultivated worldwide due to its adaptability to different climatic conditions [2]. *Jatropha curcas* cake is obtained after the extraction of its oil for use as biofuel. This waste product of refining can play a crucial role in food and nutrition [3].

The main limiting factor for the consumption of *Jatropha curcas* is the presence of toxic phorbol esters. However, non-toxic genotypes have been reported to have the potential to be used in animal diets or for human consumption [3]. From a nutritional point of view, there are reports on the chemical composition of *Jatropha curcas*, which stands out for its high crude protein content and as a source of essential amino acids. These characteristics make it a source for obtaining bioactive peptides (BPs) reported to have antimicrobial, antidiabetic, antihypertensive, hypocholesterolemic, immunomodulatory, and other properties. Bioactive peptides are generally obtained by bacterial or enzymatic hydrolysis [4]. Enzymatic hydrolysis is a simple process that presents good regioselectivity and stereoselectivity, a higher reaction speed to cleave proteins, and enzymes that are easy to inactivate. The most popular commercial enzymes are alcalase^®^ (USA, MA), protamex^®^ (USA, MA), and flavourzyme^®^ (USA, MA) [5]. This is in contrast to microbial fermentation, which is susceptible to low reproducibility with unexpected deviations in hydrolysis and inconsistency in the production of bioactive peptides industrially [5].

Marrufo-Estrada et al. [6] reported the generation of BPs from a protein isolate of *Jatropha curcas* L. (toxic genotype) by enzymatic hydrolysis of alcalase and pepsin–pancreatin, finding low-molecular-weight peptides with ACE inhibitory, antithrombotic and antioxidant activities.

The objectives of this work are to identify and quantify the ACE inhibitory, antioxidant, and antidiabetic activities of a protein isolate from *Jatropha curcas* L. var. Sevangel (no-toxic), which was hydrolyzed with alcalase and flavourzyme.

## 2. Results and Discussion

### 2.1. Protein Extraction

*Jatropha curcas* cake is mainly composed of glutelins and globulins [7]. Glutelins are soluble in acid and base solutions, while globulins are soluble in saline solutions. These factors are determinants of the solubility of the protein and, therefore, its yield [8]. The protein extract of *Jatropha* cake showed 20.44 ± 2.46% dry matter recovered, 38.75 ± 2.10% protein content, and 34.98 ± 1.92% protein yield. Specifically, the dry matter recovered, protein content, and protein yield in this work were equivalent to those Ahluwalia et al. [9] reported. *Jatropha* seed cake from Delhi, India, obtained values of 17.10% dry matter, 41.98% protein content, and 38.66% protein yield, suggesting a difference in agroecological conditions, a determinant of these parameters.

### 2.2. Proteolytic Capacity

#### 2.2.1. Analysis of Free Amino Groups

As shown in Table 1, the amino groups increased as the hydrolysis times increased. An increase was observed in the PEJA system at 2 h, reaching 4041.38 ± 50.89 ppm with no statistical difference with subsequent measurements (*p* ≥ 0.05). Therefore, under these conditions, the PEJA system obtained a higher degree of hydrolysis in a relatively short time. On the other hand, an increase was observed as time passed in the PEJF system up to 6 h, obtaining 3435.16 ± 59.31 ppm with no statistical difference with 8 h (*p* ≥ 0.05). The concentration of free amino groups depends on the enzyme used in each proteolytic system. In the case of *Jatropha curcas* L. var. Sevangel cake, the primary amino acids present were tyrosine, glutamine, histidine, and proline [3], hydrophobic amino acids where alcalase can cleave the peptide bonds of the carboxylic termini, obtaining an increase in the degree of hydrolysis compared to the PEJF system [10]. On the other hand, flavourzyme possesses activity in relation to neutral proteases, alkaline protease, leucine aminopeptidase, dipeptidyl peptidase, and amylase, taking on the role of hydrolyzing such proteins [11]. Therefore, these results suggest that *Jatropha curcas* L. var. Sevangel is a suitable substrate for alcalase and flavourzyme hydrolysis.

#### 2.2.2. Separation of Peptides by Tris-Glycine-SDS-PAGE

**Figure 1:**
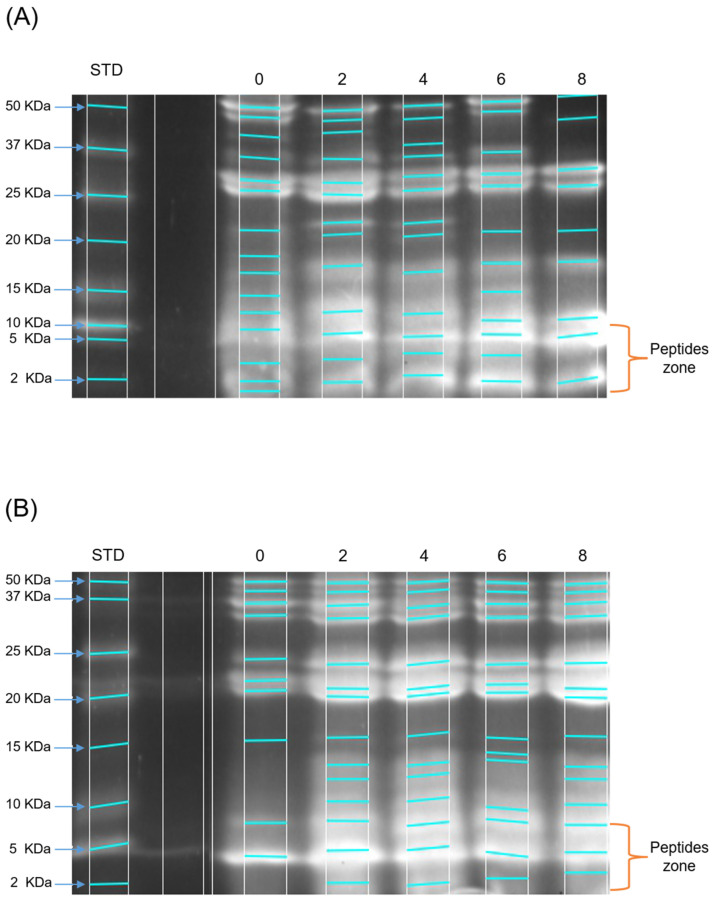
Peptide separation by Tris-Tricine-SDS-PAGE in a protein extract of *Jatropha* hydrolyzed with alcalase (**A**) and a protein extract of *Jatropha* hydrolyzed with flavourzyme (**B**). STD: peptides standard. Hydrolysis time (0–8 h). On the left side of A and B, the molecular mass range of the peptides is observed from 2 to 50 KDa. The blue lines indicate the bands detected in each lane of the electrophoresis gel. The peptide zones of interest in this study are in the range of 2–10 KDa.

Gel electrophoresis showed that the protein isolate of *Jatropha curcas* L. var. Sevangel presents a complex protein profile of 15 bands between 2 and 50 KDa (Figure 1). The large number of low-molecular-weight bands at 6 h in both hydrolysis systems suggests an extensive hydrolysis, consequently producing small peptides. The low number of electrophoretic bands at time 0 and 2 h was caused by a low degree of hydrolysis (Table 1).

**Figure 2:**
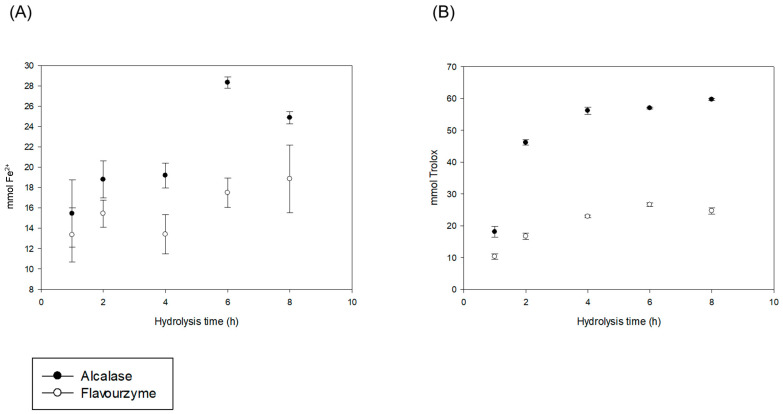
Antioxidant activity by FRAP (**A**) and DPPH (**B**) methods with alcalase and flavourzyme of hydrolysates of a protein extract of *Jatropha curcas* L. var. Sevangel. Results are expressed in 100 mL as mean ± standard error. FRAP: ferric-reducing antioxidant power; DPPH: 2,2-diphenyl-1-picrylhydrazyl hydrolysis time (0–8 h). This figure shows that the alcalase system had higher antioxidant activity than the flavourzyme system at all hydrolysis times, especially at 6 h for DPPH and 4 h for FRAP.

**Figure 3:**
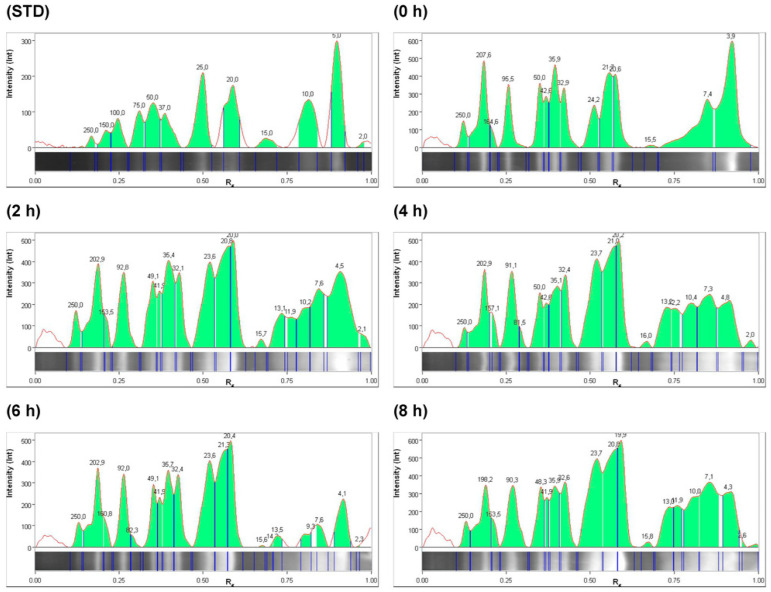
Electropherogram profile at 0 h, 2 h, 4 h, 6 h, and 8 h in *Jatropha* protein extract hydrolyzed with alcalase. STD: peptide standard. The green area in the figure represents the molecular weight concentration of the peptides; for more information see the Data Availability Statement. This figure shows an increase in the concentrations of various peptides ≤ 10 KDa for the initial time (0 h).

**Figure 4:**
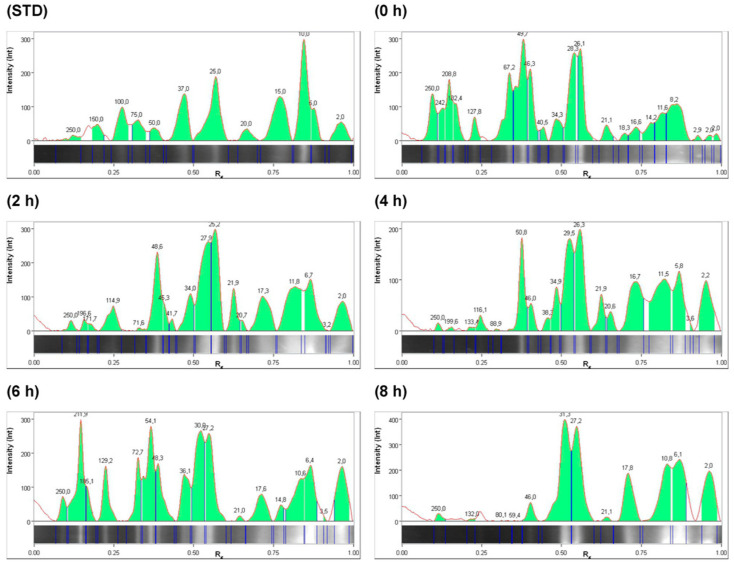
Electropherogram profile at 0 h, 2 h, 4 h, 6 h, and 8 h in *Jatropha* protein extract hydrolyzed with flavourzyme. STD: peptide standard. The green area in the figure represents the molecular weight concentration of the peptides; for more information see the Data Availability Statement. This figure shows an increase in the concentrations of various peptides ≤ 10 KDa for the initial time (0 h).

In the electrophoresis gel, bands with molecular weights related to globulins and glutelins, ranging from 15 to 50 KDa, can be observed, as reported by León-Villanueva et al. [7], and are observed to degrade as the hydrolysis time increases. This is closely related to the specificity of the enzymes used in producing the hydrolysis of the PEJ proteins. There is evidence of peptides in *Jatropha curcas* seed with a molecular weight between 693 and 1342 Da with bioactivities such as immunomodulatory, antimalarial, and antimicrobial [12]. Gel electrophoresis of PEJA and PEJF samples showed bands corresponding to peptides smaller than 10 kDa in both systems studied (Figure 1 and Figure 2). Evidence shows that peptides smaller than 10 kDa have antioxidant, antihypertensive, and antidiabetic properties [13]. The accumulation of peptides ≤ 10 KDa was higher in the alcalase system (PEJA) compared to the flavourzyme system (PEJF) (Figure 3 and Figure 4). This is associated with the high catalytic capacity due to the wide range of amino acids that can be recognized by the alcalase enzyme generating hydrolysates with small peptides. The accumulation of low-molecular-weight peptides reported in both systems (PEJA and PEJF) in the present study is higher than that reported by Marrufo-Estrada et al. [6] in a protein isolate of a toxic genotype of *Jatropha curcas* in systems with alcalase and sequential systems with pepsin–pancreatin at 60, 90 and 120 min of hydrolysis. These results suggest that the hydrolysis time, the enzyme/protein ratio and the non-toxic genotype of *Jatropha curcas* influence the degree of hydrolysis.

According to the presence of antioxidant and antihypertensive bioactivities, the peptides of interest are equal to and/or less than 10 KDa [14,15]. Therefore, during enzymatic hydrolysis, an increase in hydrolysis can be observed from the initial time. At the initial time (0 h), 2 h, 4 h, 6 h, and 8 h for the alcalase system, we reported 11.4%, 15.9%, 18.6%, 15.9%, and 27.6%, respectively, in bands ≤ 10 KDa. In the case of the flavourzyme system, 34.3%, 22.0%, 17.7%, 11.2%, and 25.1% were reported for times 0 h, 2 h, 4 h, 6 h, and 8 h, respectively (Figure 3 and Figure 4). No relationship was observed between 6 h, the time with the highest bioactivity in both systems, and the highest concentration of peptides ≤ 10 KDa.

### 2.3. Antioxidant Activity

Oxidative stress generates many health disorders, such as anti-inflammatory diseases related to tissue damage, cancer, atherosclerosis, diabetes mellitus, and neurodegenerative diseases [13]. The FRAP test is based on the reductive power of the protein hydrolysates in both systems, which was determined by the ability to reduce Fe^3+^ to Fe^2+^, which indicates the antioxidant capacity by electron donation [16]. On the other hand, the free radical-scavenging activity in the hydrolysis systems was determined using DPPH, a stable free radical, to evaluate the antioxidant activity [17].

The results are presented in Table 1 and Figure 2, where the antioxidant properties are observed in the control times. Subsequently, a linear increase occurs concerning the hydrolysis time, increasing to 6 h in both hydrolysis systems (*p* ≤ 0.05), except for DPPH (4 h) in the alcalase system. Gallegos-Tintoré et al. [18] hydrolyzed a *Jatropha curcas* protein extract with alcalase for 60 min. They observed similar behavior in terms of the antioxidant properties, obtaining an increase in antioxidant activity in the final hydrolysis phase.

These results are consistent with other studies of enzymatic hydrolysis in plant proteins, where it has been found that as the degree of hydrolysis increases, the antioxidant activities of the hydrolysates increase [13]. The PEJA samples were the hydrolysates that obtained higher antioxidant activity concerning PEJF. This is due to the high selectivity and specificity in the cleavage during protein hydrolysis, obtaining a lower molecular weight and its accumulation during hydrolysis [13]. This antioxidant activity is because peptides with a molecular weight of less than 3 kDa present high antioxidant activity [14].

Similarly, Marrufo-Estrada et al. [6] showed that the highest antioxidant capacity in a protein hydrolysate of *Jatropha curcas* (toxic genotype) was found in a sequential hydrolysis system with pepsin and pancreatin. This is related to an increased degree of hydrolysis in this system compared to the alcalase system.

### 2.4. Antihypertensive Activity

Table 1 shows the results obtained for the ACE inhibitory activity in both hydrolysis systems. PEJA showed an ACE inhibition of 81.64–91.70% with no difference between the hydrolysis times (*p* ≥ 0.05). In the flavourzyme system, the greatest increase in ACE inhibition was observed at 6 h of hydrolysis with 86.46% (*p* ≤ 0.05). These results suggest that the protein concentrate of *Jatropha curcas* L. var. Sevangel cake has antihypertensive activity per se. This differs from the data reported by Marrufo-Estrada et al. [6] in relation to a toxic genotype, as they found an increase at 2 h of hydrolysis with the alcalase system. This is possibly due to the differences between the varieties and climatic conditions among *Jatropha curcas* varieties.

On the other hand, PEJF obtained an ACE inhibition of 80.57–88.43%. However, 6 and 8 h showed an ACE inhibition of 86.46% and 88.43%, respectively, with no statistical difference (*p* ≥ 0.05). Therefore, at 6 h of hydrolysis with flavourzyme, the highest values of ACE inhibition were obtained.

The observed ACE-I inhibitory activity was higher than that reported for a *Jatropha curcas* L. hydrolysate with alcalase for 1 h (34.87%) [15]. It was even higher than that reported for amaranth hydrolysates for 4 h with alcalase and flavourzyme with 49.49 and 39.77%, respectively [19].

The high ACE inhibitory activity presented in both hydrolysis systems shows the efficiency of both enzymes in the production of antihypertensive peptides. Flavourzyme is an enzyme mixture used to produce a mixture of peptides of varying molecular weight, high molecular weight (>10 kDa), and low molecular weight (<1 kDa) [11]. Peptides with a molecular weight between 5 and 10 kDa have shown higher ACE inhibitory activity [15]. Similarly, peptides ranging from 5 to 10 kDa were observed in the electrophoresis gels in the present study (Figure 1).

Alcalase, considered a serine endopeptidase, exhibits broad selectivity and specificity in the catalyzed hydrolysis of proteins to produce peptides with hydrophobic characteristics at the C-terminal [13]. It is known that C-terminal peptides can interact at the ACE active site, resulting in ACE inhibition [6].

### 2.5. Antidiabetic Capacity

The action of the peptides generated from the hydrolysis with alcalase and flavourzyme on the inhibition of DPP-IV was evaluated (Table 1). At the beginning of the hydrolysis, values of 20.50 and 28.02% were obtained for alcalase and flavourzyme, respectively. In the flavourzyme system, it was observed that as the proteolytic activity increased, the inhibition of DPP-IV increased up to 6 h. The maximum values were reached at 6 h for the two hydrolysis systems evaluated, presenting 68.86 and 52.72% for alcalase and flavourzyme, respectively. Alcalase and flavourzyme can generate antidiabetic peptides in a protein concentrate of *Jatropha curcas* L. var. Sevangel. Hydrophobic sequences with molecular weights less than 1 kDa are highly likely to exhibit antidiabetic activity, similar to peptides with antihypertensive effects [20].

The initial values in terms of the inhibitory activity against DPP-IV agree with those of León-Villanueva et al. [7], who reported a protease with antidiabetic activity in stored *Jatropha curcas* cake.

The generation of bioactive peptides with alcalase and flavourzyme that exhibit inhibitory activity against DPP-IV has previously been reported in other hydrolyzed grains, such as lupine, brewer’s spent grain, oat protein, soybean paste, and rye protein [21,22,23,24,25]. Sharma et al. [26] obtained a DPP-IV inhibition of 51.51 ± 0.28% from soluble corn distillates with sequential hydrolysis by alcalase and flavourzyme. These values are lower than those reported at 6 h with alcalase and similar to the values obtained with flavourzyme in *Jatropha curcas*. *Jatropha curcas* may be a promising source for obtaining bioactive peptides with antidiabetic activity.

## 3. Materials and Methods

### 3.1. Sample

Seeds of *Jatropha curcas* L. var. Sevangel were provided in January 2023 by the Centro de Desarrollo de Productos Bióticos (CEPROBI-IPN) located in Yautepec, Morelos, Mexico. After extracting the oil, the seeds were air-dried and mechanically compressed (7111.5 psi, 80 °C). The resulting residues were ground, sieved, and later defatted using the Bligh–Dyer methodology [27] to obtain *Jatropha curcas* cake. Subsequently, the sample was stored in a container at room temperature until protein extraction.

### 3.2. Protein Extraction

Protein extraction was performed following the methodology of Ahluwalia et al. [9] with the following conditions: temperature of 60 °C, solubilization pH of 11.0, precipitation pH of 4.41, and an extraction time of 0.78 h. The protein extract of *Jatropha curcas* L. var. Sevangel was freeze-dried (0.045 mBar; −52 °C) and stored at 4 °C until use. The freeze-dried extract was referred to as PEJ. The percent dry matter recovered (Equation (1)) and protein yield (Equation (2)) were calculated from the freeze-dried sample of PEJ according to the following formulas (Equations (1) and (2)):

(1) $$Dry\;matter(\%)=\frac{Dry\;matter\;weight\;of\;protein\;precipitate}{Initial\;seed\;weight\;on\;dry\;basis}\times 100$$

(2) $$Protein\;yield(\%)=\frac{DME\left(g\right)\times protein\;content\;extract(\%)}{ICW\left(g\right)\times cake\;protein\;content(\%)}\times 100$$

where:

DME = Dry matter extract

ICW = Initial cake weight

### 3.3. Enzymatic Hydrolysis

Enzymatic hydrolysis was performed following the methodology of Islas-Martínez et al. [25]. Firstly, 10 g of PEJ and 0.1% xanthan gum (*m*/*v*) was taken and diluted in phosphate buffer (0.1 M, pH = 7.5) and then pasteurized (90 °C for 10 min). The PEJ was hydrolyzed by the alcalase (>2.4 U/g, Hanson units; Sigma-Aldrich, USA, MA) (PEJA) and flavourzyme (>500 U/g; Sigma-Aldrich, USA, MA) (PEJF) systems at a total protein/enzyme mass ratio of 100:2.5 in both cases. The PEJ was hydrolyzed for 8 h at 60 °C and 130 rpm with oscillating agitation for both reaction systems (alcalase and flavourzyme), and samples were taken every 2 h. Then, each collected sample was heated at 85 °C for 10 min to stop the reaction. Finally, the samples were centrifuged at 10,000 rpm for 10 min at 4 °C, and the supernatant was separated and stored at −18 °C for use in subsequent analyses.

### 3.4. Proteolytic Capacity

The proteolytic capacity was determined by the TNBS technique, and the peptides that were released in the enzymatic hydrolysis were separated using Tris-Glycine polyacrylamide gel electrophoresis (SDS-Tris-Glycine-PAGE).

#### 3.4.1. Free Amino Groups by the TNBS Method

Determination of the hydrolysis degree was performed by the 2,4,6-trinitrobenzene sulfonic acid (TNBS) test according to the methodology of Sashidhar et al. [28], with some modifications. The PEJ samples (0.250 mL) were mixed with 2 mL of 0.21 M phosphate buffer solution at pH 8.2 and 2 mL of 0.1% TNBS reagent (Sigma-Aldrich, St. Louis, MO-USA). The resulting mixes were incubated at 50 °C for 60 min, protected from light. Four mL of 0.1 M HCl was added to stop the reaction, and its absorbance was read at 340 nm. The concentration of free amino groups was obtained from a calibration curve performed with glycine at concentrations of 0–200 ppm.

#### 3.4.2. Tris-Glycine Polyacrylamide Gel Electrophoresis (Tris-Glycine-SDS-PAGE)

The method proposed by Pérez-Escalante et al. [29] was performed. Electrophoresis was performed on a 15% separating T-gel and 4% for a concentration T-gel from a 30% T-solution (acrylamide/bisacrylamide 37.5:1 and 2.7% cross-linker, Bio-Rad, Hercules, CA, USA) and analyzed with Gel-Doc EZ (Image Lab 6.1) imaging system software (Bio-Rad, Hercules, CA, USA).

### 3.5. Antioxidant Activity

The antioxidant activity was evaluated using two different methods, as described below.

#### 3.5.1. Ferric-Reducing Power

The antioxidant activity was evaluated using Benzie and Strain’s methodology [16]. A FRAP preparation was obtained at a 10:1:1 ratio of 0.3 M sodium acetate buffer at pH 3.6, TPTZ (Sigma-Aldrich, St. Louis, MO, USA), and ferric chloride at 20 mM. Subsequently, 250 μL of PEJA and PEJF samples were mixed with 1 mL of FRAP solution to 10 mL volumetric capacity with distilled water and incubated at 37 °C for 15 min. The absorbance was measured at 593 nm, and the results were compared with an iron (II) sulfate calibration curve at 200 mM. The antioxidant activity was expressed as micromole equivalents of iron (II) sulfate per 100 mL (mmol E Fe II/100 mL).

#### 3.5.2. Radical Scavenging Properties by DPPH

The antiradical activity was determined by Brand-Williams et al.’s [17] technique. A methanolic solution of DPPH was prepared (0.1 mM dissolved in methanol). Firstly, 100 μL of the PEJA or PEJF sample was added to 2.9 mL DPPH solution and kept for 50 min. Finally, the absorbance of the supernatant was measured at 515 nm and expressed as mg Trolox equivalents/L (mg ET/100 mL).

### 3.6. Antihypertensive Activity

The antihypertensive activity was assessed by the angiotensin-converting enzyme (i-ACE) inhibitory effect, according to Cushman et al. [30]. First, 200 μL of substrate hippuryl-histidyl-leucine (HHL) (Sigma-Aldrich, St. Louis, MO, USA) and 20 μL of rabbit lung angiotensin-converting enzyme reconstituted in buffer to achieve a concentration of 0.1 U/mL (EC 3. 4 15.1, 0.1 U/mg; Sigma-Aldrich, St. Louis, MO, USA) were added to 80 μL of 5 mM sodium borate solution, at pH 8.2, in an of 0.3 M sodium chloride solution to obtain a final concentration of 5 mM of the substrate. Subsequently, 80 μL of the PEJA or PEJF sample replaced the buffer and was mixed with 200 μL of HHL and 20 μL of ACE. The reaction was carried out for 80 min at 37 °C, and the enzyme was inactivated with 1 mL of 0.1 M HCl. The hippuric acid formed was extracted with 1.7 mL ethyl acetate and diluted with deionized water to measure its absorbance at 220 nm. The organic extraction was performed three times, and 800 µL of the organic layer was separated and evaporated by heating at 80 °C for 1 h in the same dry bath. The extracted hippuric acid was reconstituted with 500 µL of deionized water. The latter volume was mixed with 300 µL of pyridine and 150 µL of benzene sulfonyl chloride, and the absorbance at 410 nm was recorded in a UV–vis spectrophotometer. The inhibitory activity of ACE was calculated by the following formula (Equation (3)):

(3) $$Inhibitory\;activity(\%)=\frac{A_{C}-A_{S}}{A_{C}-A_{B}}\times 100$$

where:

A_C_ = Hippuric acid formed during the action of ACE without an inhibitor

A_B_ = Unreacted hippuryl-histidyl-leucine that was extracted with ethyl acetate

A_S_ = Hippuric acid formed after the action of ACE in the presence of an inhibitory substance

### 3.7. Antidiabetic Capacity

The antidiabetic capacity was determined by the DPP-IV inhibitory activity [31], with some modifications. A 100 μL sample of PEJA or PEJF added to 100 μL of Gly-Pro-p-nitroanilide at 1.6 mM in Tris-HCl buffer (0.1 M, pH = 8) was pre-incubated at 37 °C in a dry bath for 10 min, and added then to 200 μL of DPP-IV diluted in Tris-HCl (0.01 U/mL). After 60 min, the reaction was stopped by adding 400 μL of 0.1 M potassium carbonate solution. Finally, the absorbance was measured at 405 nm, and the DPP-IV inhibition (DI) was calculated by the following equation (Equation (4)):

(4) $$Inhibitory\;DPP-IV\left(\%\right)=\frac{A_{100}{-(A}_{S}-A_{SC})}{A_{100}}\times 100$$

where:

A_100_ = Absorbance at 405 nm of the enzyme reaction without hydrolysate.

A_S_ = Absorbance at 405 nm of the enzymatic reaction with hydrolysate.

A_SC_ = Absorbance at 405 nm of hydrolysate where the enzyme and substrate were replaced by Tris-HCl buffer pH = 8 and potassium carbonate.

### 3.8. Statistical Analysis

All the experiments were performed in triplicate and analyzed by one-way ANOVA. Tukey’s contrast test determined significant differences at a significance of 0.05. All the statistical tests were analyzed with Statgraphics Centurion XVI.I software.

## 4. Conclusions

The enzyme systems proposed for a non-toxic genotype of *Jatropha* (*J. curcas* L var. Sevangel) were shown to have antioxidant, antihypertensive, and antidiabetic bioactivity. However, the best results were obtained using alcalase. Previous studies highlighted *J. curcas* as a source of peptides showing antioxidant and antihypertensive activity. The generation of bioactive peptides from the *Jatropha curcas* protein concentrate showed its nutritional and pharmaceutical potential for treating chronic degenerative diseases, such as cancer, diabetes, and hypertension. However, this is the first report of its antidiabetic activity. Regarding multi-bioactivity, the alcalase hydrolysate performed better than the flavourzyme hydrolysate and the cereals reported in other studies. These findings are important for obtaining the sequencing of peptides with multi-bioactivities. For all these reasons, the by-product of *Jatropha curcas* is emerging as a functional food due to its high protein content, which can contribute to treatments for diabetes, blood hypertension, and the reduction of free radicals.

## Figures and Tables

**Table 1:** Hydrolysis degree and bioactivities (antioxidant, antihypertensive, and antidiabetic) with alcalase and flavourzyme of *Jatropha curcas* L. var Sevangel protein extract hydrolysates.

Time (h)	Hydrolysis Degree (ppm)	FRAP (mmol Fe^2+^)	DPPH (mmol Trolox)	ACE-I Inhibition (%)	DPP-IV Inhibition (%)
Alcalase					
0	1277.25 ± 119.26 ^a^	15.45 ± 3.31 ^a^	18.11 ± 1.71 ^a^	87.34 ± 0.62	20.50 ± 1.02 ^a^
2	4041.38 ± 50.89 ^bc^	18.80 ± 1.82 ^a^	46.17 ± 0.86 ^b^	80.57 ± 5.25	40.87 ± 2.04 ^b^
4	3802.28 ± 267.04 ^b^	19.19 ± 1.23 ^a^	56.17 ± 1.14 ^c^	83.19 ± 4.63	32.07 ± 0.19 ^ab^
6	4437.48 ± 345.70 ^c^	28.33 ± 0.55 ^b^	57.01 ± 0.34 ^cd^	81.64 ± 0.03	68.86 ± 8.27 ^c^
8	4514.77 ± 163.79 ^c^	24.87 ± 0.60 ^b^	59.70 ± 0.30 ^d^	91.70 ± 1.24	43.10 ± 2.57 ^b^
Flavourzyme					
0	859.86 ± 13.28 ^a^	14.77 ± 1.44 ^a^	10.35 ± 0.90 ^a^	81.00 ± 0.93 ^a^	28.02 ± 1.75 ^a^
2	2090.29 ± 83.90 ^b^	14.68 ± 0.12 ^a^	16.72 ± 0.95 ^b^	80.79 ± 0.62 ^a^	39.59 ± 3.35 ^b^
4	2901.40 ± 73.30 ^c^	14.51 ± 0.12 ^a^	22.95 ± 0.37 ^c^	80.57 ± 0.31 ^a^	24.59 ± 0.84 ^a^
6	3435.16 ± 59.31 ^d^	18.26 ± 0.84 ^b^	26.64 ± 0.52 ^d^	86.46 ± 1.85 ^b^	52.75 ± 2.23 ^c^
8	3843.34 ± 333.33 ^d^	20.73 ± 0.96 ^b^	24.70 ± 1.00 ^cd^	88.43 ± 0.31 ^b^	50.17 ± 1.51 ^c^

FRAP: ferric-reducing antioxidant power; DPPH: 2,2-diphenyl-1-picrylhydrazyl; ACE-I: angiotensin I-converting enzyme; DPP-IV: dipeptidyl peptidase IV. The DPPH and FRAP results are expressed in 100 mL of hydrolysate. All the results are expressed as the mean ± standard deviation. Different letters between rows for each enzyme system indicate significant differences (*p* ≤ 0.05). The table shows that maximum values obtained for the bioactivities of both the alcalase and flavourzyme systems correspond to 6 h of hydrolysis, except for DPPH (4 h) in the alcalase system. There was no statistical difference in ACE inhibition for the alcalase system.

## Data Availability

The data provided from the gel electrophoresis (image reports and electropherograms) can be found at the following link: https://drive.google.com/drive/folders/1Wx5FI4KXogn3vZ70X651y8OjsZRmlfsO?usp=sharing (accessed on 23 May 2024).
